# An Efficient Machine Learning-Based Emotional Valence Recognition Approach Towards Wearable EEG

**DOI:** 10.3390/s23031255

**Published:** 2023-01-21

**Authors:** Lamiaa Abdel-Hamid

**Affiliations:** Department of Electronics & Communication, Faculty of Engineering, Misr International University (MIU), Heliopolis, Cairo P.O. Box 1 , Egypt; lamiaa.a.hamid@miuegypt.edu.eg

**Keywords:** classification, EEG, emotion recognition, prefrontal channels, time and frequency features

## Abstract

Emotion artificial intelligence (AI) is being increasingly adopted in several industries such as healthcare and education. Facial expressions and tone of speech have been previously considered for emotion recognition, yet they have the drawback of being easily manipulated by subjects to mask their true emotions. Electroencephalography (EEG) has emerged as a reliable and cost-effective method to detect true human emotions. Recently, huge research effort has been put to develop efficient wearable EEG devices to be used by consumers in out of the lab scenarios. In this work, a subject-dependent emotional valence recognition method is implemented that is intended for utilization in emotion AI applications. Time and frequency features were computed from a single time series derived from the Fp1 and Fp2 channels. Several analyses were performed on the strongest valence emotions to determine the most relevant features, frequency bands, and EEG timeslots using the benchmark DEAP dataset. Binary classification experiments resulted in an accuracy of 97.42% using the alpha band, by that outperforming several approaches from literature by ~3–22%. Multiclass classification gave an accuracy of 95.0%. Feature computation and classification required less than 0.1 s. The proposed method thus has the advantage of reduced computational complexity as, unlike most methods in the literature, only two EEG channels were considered. In addition, minimal features concluded from the thorough analyses conducted in this study were used to achieve state-of-the-art performance. The implemented EEG emotion recognition method thus has the merits of being reliable and easily reproducible, making it well-suited for wearable EEG devices.

## 1. Introduction 

Emotion artificial intelligence (AI), also known as affective computing, is the study of systems that can recognize, process, and respond to the different human emotions, thereby making people’s lives more convenient [1]. Emotion AI is an interdisciplinary field that combines artificial intelligence, cognitive science, psychology, and neuroscience. In 2019, the emotion AI industry was worth about 21.6 billion dollars, and its value was predicted to reach 56 billion dollars by the year 2024 [2].

**Figure 1:**
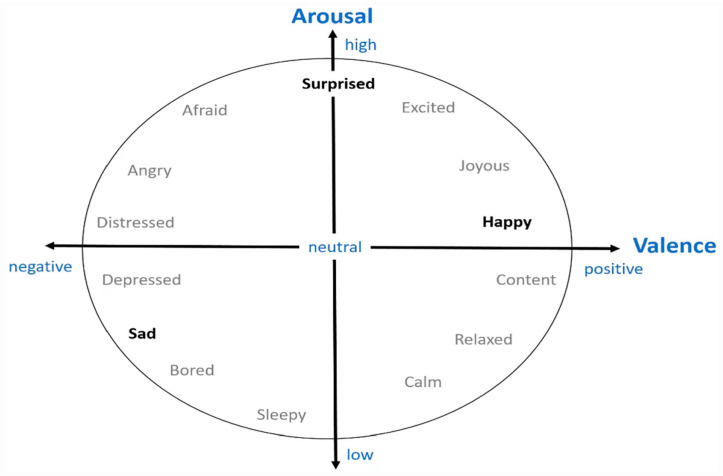
Valence-arousal model [6].

Emotions are mental states created in response to events occurring to us or in the world around us. A large body of research since the 1970s showed that basic emotions, such as happiness, sadness, and anger are similarly expressed among different cultures [3]. James Russell, a renowned American psychologist, suggested a dimensional approach in which all human emotions could be expressed in terms of valence and arousal [4]. Valence refers to the extent to which an emotion is pleasant (positive/happy) or unpleasant (negative/sad), whereas arousal (intensity) refers to the strength or mildness of a given emotion (Figure 1). Russell’s valence-arousal model is very popular owing to its simplicity and efficacy, both which lead to it being widely adopted in emotion AI systems [5]. 

Emotions can be detected from a person’s facial expressions and tone of speech. Although these methods were previously considered for automatic emotion recognition [7,8], they both have the limitation of being easily manipulated by a person to hide his/her true emotions [5,9]. Electroencephalography (EEG) is a non-invasive technique that can measure spontaneous human brain activity while providing excellent temporal resolution yet limited spatial resolution [10]. EEG can thus provide a reliable method to detect and monitor true, unmanipulated human emotions. EEG-based emotion recognition has been successfully implemented in various applications including (1) education: to measure student engagement, (2) health: to diagnosis psychological diseases, and (3) emotion-based music players: to provide a more engaging experience [11]. 

**Figure 2:**
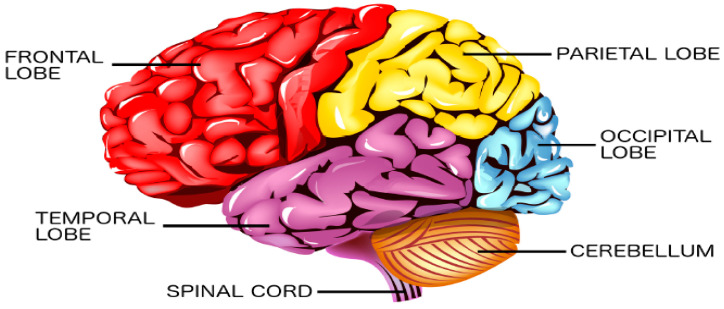
The cerebral cortex divided into the frontal, temporal, parietal, and occipital lobes [13].

**Figure 3:**
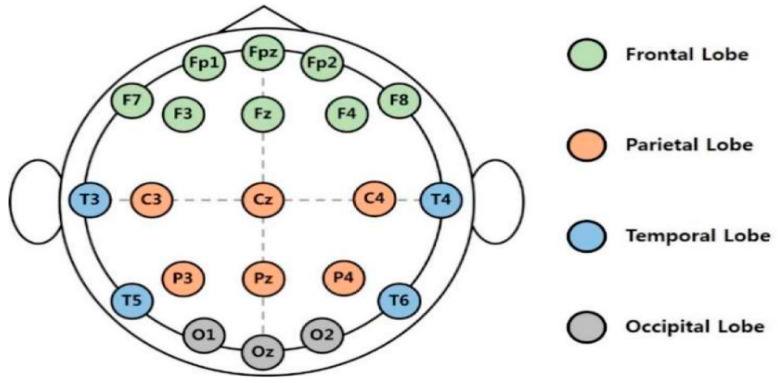
The international 10/20 system for electrode placement [14].

The cerebral cortex is the outermost layer of the brain that is associated with the highest mental capabilities. The cerebral cortex is traditionally divided into four main lobes which are the frontal (F), parietal (P), occipital (O), and temporal (T) (Figure 2). Each brain lobe is typically associated with certain functions, yet many activities require the coordination of multiple lobes [12]. The frontal lobe is responsible for cognitive functions such as emotions, memory, decision making, and problem solving, as well as voluntary movement control. The parietal lobe process information received from the outside world such as that related to touch, taste, and temperature. The occipital lobe is primarily responsible for vision, while the temporal lobe is responsible for understanding language, perception, and memory. EEG depicts the brain’s neuron activity in the different lobes through measuring the electrical voltage at the scalp. For an adult, this voltage is typically in the range of 10–100 µV. The 10/20 system is an internationally recognized EEG electrode placement method that divides the scalp into 10% and 20% intervals. The main EEG channels in the international 10/20 system are illustrated in Figure 3. Each channel is annotated with a letter and a number to identify the specific brain region and hemisphere location, respectively.

**Figure 4:**
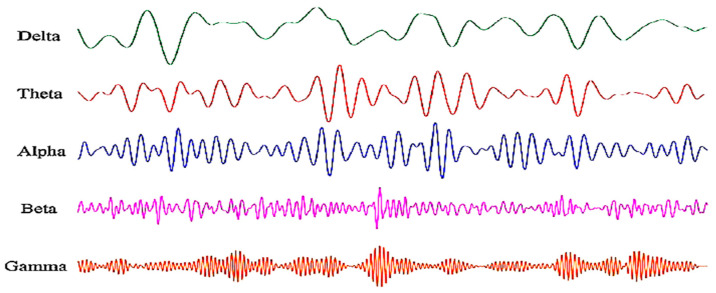
Samples from delta, theta, alpha, beta, and gamma brain waves [28].

EEG signals are typically decomposed into five basic frequency bands which are the delta (*Δ*), theta (*θ*), alpha (*α*), beta (*β),* and gamma (*δ*) bands (Figure 4). Each frequency band is associated with a different type of brain activity [15,16,17]. Delta and theta are the two slowest brain waves often occurring whilst sleeping and during deep meditation. Specifically, delta waves are more dominant in deep restorative sleep (unconsciousness), whereas theta waves are related to light sleep, daydreaming, praying, and deep relaxation (subconsciousness). Both waves were also detected in cognitive processing, learning, and memory [17,18]. Alpha, beta, and gamma brain waves are on the other hand associated with consciousness. Alpha are the dominant brain waves of normal adults occurring when one is calm and relaxed while still being alert. Beta waves are produced throughout daily activities performed in attentive wakefulness. Gamma are the fastest waves linked to complex brain activities requiring high level of thought and focus, for example problem solving. Table 1 summarizes the five different brain wave bands and their associated psychological states. Brain wave frequency bands are typically used to extract meaningful emotion-related features [17].

**Figure 5:**
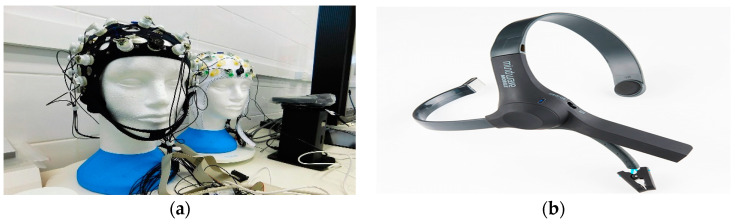
(**a**) Conventional lab EEG headset [29] versus (**b**) wearable headset from NeuroSky [30].

Historically, EEG equipment has been highly complicated and bulky, restricted to the monitoring of stationary subjects by highly trained technical experts within controlled lab settings [19]. Recently, enormous effort has been exerted to develop wearable EEG handsets that are reliable, affordable, and portable, by that overcoming the limitations of conventional EEG headsets (Figure 5). Wearable EEG headsets allow for the long-term recording of brain signals while people are unmonitored, out of the lab, and navigating freely. Furthermore, EEG signals collected by the wearable headsets can be easily sent to a computer or mobile device for storage, monitoring, and/or data processing. Wearable EEG devices thus allow for the development of many clinical and non-clinical applications that were never previously possible. For example, wearable EEG has been shown to be effective for stroke [20], seizure [21], and sleep [22] remote monitoring by medical experts. EEG signals from wearable headsets can also be used for the development of brain-controlled-interface (BCI) applications such as car driver assistance [23], as well as wheelchair control for people with disability [24]. In addition, individuals can use EEG to improve their productivity and wellness via monitoring their moods and emotions [25]. However, extracting meaningful information using few EEG channels in order to reduce the computational complexity of wearable headsets is still an ongoing challenge [26,27].

In the present study, a subject-dependent emotional valence recognition algorithm is introduced that is intended for wearable EEG devices. The contributions of this work are as follows:

- Only the difference signal between the frontal Fp1 and Fp2 channels was considered for feature extraction.
- Simple statistical features were explored (Hjorth parameters, zero-crossings, and power spectral density), all which share the merit of having low computational complexity.
- Several analyses were made to determine the frequency band, time slot, and features most suitable for reliable EEG-based valence detection.
- The presented valence recognition algorithm outperformed several state-of-the-art methods with the added advantages of requiring only two EEG channels, a single frequency band, as well as only two simple statistical features, thus making it suitable for integration within wearable EEG devices.

## 2. Literature Review

**Figure 6:**
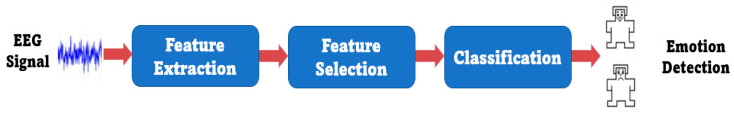
Emotion AI system diagram.

Emotion AI systems generally rely on handcrafted and/or automatic extraction of meaningful features for the classification of the different human emotional states (Figure 6). In this section, the different types of EEG-based features commonly used for emotion recognition are introduced followed by a summary of the most widely used classifiers for emotion recognition. Next, state-of-the-art EEG-based emotion detection methods from literature are presented, indicating the considered EEG channels, frequency bands, features, and the classifier, as well as the performance results.

### 2.1. EEG Features

EEG-based emotion recognition features can be categorized based on the domain from which they are computed into four different types which are as follows [31]:

**(1)**  **Time-domain (spatial) features** are handcrafted features that are extracted from the EEG time-series signal. They can be computed directly from the raw EEG signal or from the different frequency bands separated with the aid of bandpass filters. Time-domain features comprise simple statistical features [32,33,34] such as the mean, standard deviation, skewness, and kurtosis. In addition, they include more complex features such as the Hjorth parameters [5,32,35,36,37,38,39,40,41], High Order Crossings (HOC) [5,33,38,40,42], Fractal Dimensions [43,44,45], Recurrence Quantification Analysis (RQA) [46,47], in addition to entropy-based features [5,34,35,45,48].
**(2)**  **Frequency-domain features** are also handcrafted features, yet they are computed from the EEG signal’s frequency representation. The Fast Fourier transform (FFT) and Short-time Fourier Transform (STFT) are typically used to acquire the frequency-domain signal from the EEG waves. Frequency-based features allow for the deeper understanding of the signal by considering its frequency content. Frequency-domain features include the widely used power spectral density (PSD) [33,35,39,49,50,51], as well as rational asymmetry features (RASM) [32,34,39,52,53]. Statistical features such as mean, median, variance, skewness, and kurtosis are also commonly computed in the EEG’s the frequency domain, as well as the relative powers of the various frequency bands [54].
**(3)**  **Time-frequency domain features** are handcrafted features extracted from sophisticated time-frequency signal representations. Wavelet transform (WT) is a powerful tool that can decompose a signal into different subbands by applying a series of successive high and low frequency filters. WT has the advantage of being localized in both time and frequency. It can thus be used to divide the EEG signal into the delta, theta, alpha, beta, and gamma subbands from which wavelet time-frequency features can be directly computed for emotion classification. Wavelet features typically include simple statistical measures such as mean, standard deviation, skewness, kurtosis, energy, and entropy [9,32,39,53,55,56,57].
**(4)**  **Deep features** refer to those features that are automatically extracted in an end-to-end manner using one or more deep networks. Deep features have been gaining increased popularity and are being used either solely or alongside handcrafted (traditional) features in emotion AI [58]. Inputs to the deep networks can be the raw EEG signal [59,60], traditional features [61], or images that are obtained either from the EEG signal’s Fourier Transform (spectrograms) or Wavelet Transform (scalograms) [62,63,64,65]. In addition, the deep networks used for the feature extraction can be directly utilized or initially pretrained (transfer learning) to enhance performance.

Handcrafted (traditional) features have been widely implemented in the design of reliable EEG-based emotion AI systems. Time-domain features have the merit of being easy to implement while efficiently extracting relevant information from the EEG signals. Specifically complex time domain features such as Hjorth parameters and High Order Crossings were shown to give reliable results in EEG emotion recognition [31]. Frequency-domain features have also been widely implemented for EEG emotion recognition due to their efficient performance, yet they have the disadvantage of missing temporal information. Wavelet-domain features have the advantages of being localized in time and frequency allowing for extraction of simple yet meaningful features from the signal. A limitation of the wavelet-based features is the selection of a suitable mother wavelet [31]. Most EEG-based emotion recognition approaches thus combine different types of features for consistent performance. Several traditional classifiers were implemented in literature to classify the handcrafted features from which some of the most popular are support vector machine (SVM), k-nearest neighbor (kNN), random forests (RF), naïve Bayes (NB), and gradient boosted decision trees (GBDT) [66].

As for deep learning approaches, convolutional neural networks (CNNs), deep belief networks (DBN), and long short-term memory networks (LSTMs) among others have been used for feature extraction in emotion AI systems. In addition, pretrained readily available CNNs, such as GoogleNet, were widely used in literature as they tend to give reliable performance without requiring enormous data for training. A SVM classifier as well as sigmoid/softmax activation functions are then typically used at the network’s final stage for emotion classification. Deep EEG emotion recognition methods, however, have the limitation of requiring a huge amount of data for their proper training in comparison to traditional methods [54].

### 2.2. Previous Literature

Several public EEG emotion datasets were introduced including DEAP [67], SEED [68,69], MAHNOB-HCI [70], and DREAMER [71]. Few works also report results using their own private self-generated datasets [51]. DEAP is currently considered the benchmark dataset in EEG-based emotion detection being the most widely used public EEG emotion dataset in the literature, mostly owing to it having the largest number of observations per subject [72].

EEG emotion recognition approaches can be divided into subject-dependent and subject-independent [46,73]. Subject-dependent methods train a separate model for each subject within the dataset. Subject-independent methods train a single model using data from all or some of the subjects within the considered dataset [74]. Recent papers comparing subject dependent and independent approaches showed that the former consistently gave 5–30% higher performance depending on the implemented approach. Such results are mainly due to the discrepancy between subjects related to how they feel and express their emotions [75]. For example, Nath et al. [73] have observed that EEG signals from a specific subject were somewhat similar yet significantly varied across different subjects, even when the same stimulus was considered. In addition, Putra et al. [75] found that different subjects varied in their response to valence stimuli, with some subjects being more responsive than others [75]. Subject-dependent approaches are thus better suited for reliable personalized emotion AI applications with wearable EEG [64].

Table 2 summarizes some of the recent EEG emotion recognition approaches using the benchmark DEAP dataset. For each research paper, the summary indicates the utilized (1) EEG channels, (2) frequency bands, (3) feature types: time—frequency—wavelet—deep features, (4) classifier, (5) experimental approach: subject-dependent (*dep*.)—subject-independent (*indep*.), as well as the (6) accuracies (*Acc*.) reported for valence (*val*.) and arousal (*arl*.) emotion recognition. For the subject independent emotion recognition methods, reported accuracies are for the experiments performed considering the complete dataset. As for the subject dependent methods, reported accuracies are the average of the experiments repeated for all the subjects in the dataset. The summarized literature review shows that subject-dependent (personalized) approaches that adopted deep learning methods, gave accuracies that were higher than 90% for both valence and arousal. However, subject-dependent approaches relying solely on traditional methods scarcely resulted in accuracies that exceeded 75%. Another limitation observed in previous literature is that most methods consider many or all EEG channel electrodes and/or frequency bands which can lead to high computational overhead with minimal, if any, performance improvement.

In the present study, a subject-dependent approach is adopted for valence (happy/sad) emotion classification intended for personalized emotion AI applications with wearable EEG. Since several previous studies showed that the frontal channels are the most relevant for EEG-based emotion recognition [33,39,40,53,83], only the Fp1 and Fp2 channels were considered for emotion recognition. The widely used DEAP benchmark dataset was considered for its reliability, as well as to facilitate comparison to previous approaches. Time and frequency EEG features were extracted from a single time series related to the Fp1 and Fp2 channels which are the Hjorth parameters, zero-crossings, and PSD.

Happiness and sadness emotions (valence) have been reported to dramatically affect the theta, alpha, and beta waves of the frontal channels [84]. Interestingly, the delta [85], alpha [86], and gamma [87,88] waves of the frontal channels were also shown to be individually useful for EEG-based emotion recognition. Several analyses were thus performed in this work to determine the frequency bands most suitable for valence detection considering the different computed features. In addition, performance was observed when the compete EEG signal was considered for feature computation in comparison to when only a short segment was utilized. The aim of the performed analyses was to find the most suitable feature set that would achieve superior performance comparable to state-of the-art methods, all while requiring minimal computational overhead. Primarily, only the sixteen strongest emotions (eight happiest and eight saddest) were considered in the analyses in order to assure significant discrepancy between the emotions. Then, the complete DEAP dataset was utilized for the final experimentations concerning binary and multiclass valence classifications, as well as for comparison to previous literature.

## 3. Methods

### 3.1. Dataset

DEAP is a public audio-visual stimuli-based emotion dataset [67] that was collected from 32 subjects. For emotion recognition, the use of audio-visual stimuli guarantee higher valence intensity is experienced with respect to visual stimuli (pictures) [89]. The subjects ages ranged between 19 and 37, with an average of 26.9 years. Each subject watched 40 one-minute music videos intended to elicit different emotions. These one-minute videos were extracted from long-version music videos to include maximum emotional content. EEG signals from thirty-two electrodes placed according to the international 10/20 system were recorded at a sampling rate of 512 Hz then downsampled to 128 Hz. Each electrode recorded 63 s EEG signal, with a 3s baseline signal before the trial. The 3 s baseline was ignored here as previously performed in [58,76,77,90].

After watching each video, participants performed a self-assessment of their emotional states of valence, arousal, liking, and dominance on a continuous scale from 1 to 9. Only valence was considered in the present study which would be useful for personalized medical applications as well as in emotion-based entertainment content. The valence scale ranges from sad to happy with ratings closer to one representing low valence (sad), whereas ratings closer to nine indicating high valence (happy). For the binary classification experiments, a threshold (*thresh*.) of five was considered to separate the low and high valence classes as commonly performed in many other works such as Refs. [58,60,61,73,74,76,77,91,92,93,94]. This threshold value is typically chosen to overcome the class imbalance issue in the DEAP dataset [64,67]. As for the three-class classifications, thresholds of three and six were considered to divide the dataset into low valence (sad), mid-range (neutral), and high valence (happy).

### 3.2. Channel Selection

The international 10/20 system includes several electrode placement markers applied to detect the brain waves from the different brain lobes. In deep learning approaches where it is basically the network’s task to extract meaningful features from the data, it is common to input all the EEG channels to the network for emotion recognition [59,60,94]. Nevertheless, several studies have shown that considering all EEG channels can be redundant and that extracting features from a few significant channels can results in reliable performance with the added advantage of reduced computational complexity [35,53,55]. 

For wearable EEG headsets, requiring only one or two EEG channels can substantially reduce the hardware complexity thus facilitating its usage in non-laboratory settings, as well as reducing its overall cost, all which would make it more attractive to day-to-day consumers [35,53,95]. From the different brain lobes, the frontal lobe is the one most associated with emotion recognition using EEG signals [5]. Specifically, several studies have shown that features calculated from the prefrontal brain region (Fp1-Fp2) result in best performance as compared to other brain areas [35]. Mohammadi et al. [55] more specifically showed that the Fp1-Fp2 channel pair resulted in highest accuracies in comparison to other frontal channel pairs, and that combining all the frontal channels resulted in a somewhat enhanced performance. Interestingly, Wu et al. [53] found that not only did Fp1-Fp2 result in the highest accuracies in comparison to the other frontal channels, but that solely using Fp1-Fp2 resulted in similar performance to the case when features from four or six frontal channels were combined. The Fp1-Fp2 channel pair was thus chosen in this study for valence-related feature extraction. 

Previous research has shown that positive emotions are associated with left frontal activity, whereas negative emotions are associated with right frontal activity [96,97]. Symmetric channel pairs from the left and right brain hemispheres were thus commonly considered in literature by being either subtracted or divided in order to create a single wave from which relevant features were calculated [61,98,99]. In the present study, the EEG features were extracted from a single time series signal computed as the difference between the Fp1 and Fp2 channels in order to measure the asymmetry in brain activity due to the valence emotional stimuli [67]. 

### 3.3. EEG Band Separation

Five different third order Butterworth band-pass filters were implemented to separate the delta (2–4 Hz), theta (4–8 Hz), alpha (8–12 Hz), beta (12–30 Hz), and gamma (30–60 Hz) frequency bands (Table 1). The Butterworth filter has been previously used for the EEG bands separation owing to its flat response, simplicity, and efficiency [5,40].

### 3.4. Feature Extraction

Both time and frequency domain EEG features were initially computed from all the frequency bands (delta–theta–alpha–beta–gamma). Next, feature analysis was performed to determine which features were more suitable for valence emotion recognition, as well as the most relevant frequency band for feature extraction.

**A.**  **Hjorth Parameters**

**Figure 7:**
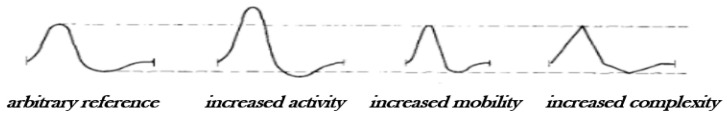
Characteristic changes in an arbitrary reference signal, illustrating their relation to the different Hjorth parameters [100].

Hjorth parameters [100] were introduced by Bo Hjorth in 1970 to represent several signal statistical properties (Figure 7). Hjorth parameters have been successfully used in various EEG emotion recognition research [5,32,35,36,37,38,39,40]. The three Hjorth parameters are activity (variance), mobility, and complexity given by the following equations:

(1) Activity=varaince (y(t))

(2) $$Mobility=\sqrt{\frac{activity\left(dy\left(t\right)/dt\right)}{activity\left(y\left(t\right)\right)}}\;$$

(3) $$Complexity=\sqrt{\frac{mobility\left(dy\left(t\right)/dt\right)}{mobility\left(y\left(t\right)\right)}}\;$$

**B.**  **Zero-Crossings**

The zero-crossings of a signal are the number of times the signal intercepts the horizontal x-axis thus changing signs. Zero crossings are used to measure the oscillating property of a signal indicating the degree of excitation within a specific frequency band.

**C.**  **Power Spectral Density**

Power spectral density (PSD) is among the most widely implemented EEG features for emotion recognition [72]. PSD describes the average signal power over its frequency bands. To obtain the PSD, the amplitude of the FFT is multiplied by its complex conjugate which is then summed to get the total power. 

## 4. Results

**Figure 8:**
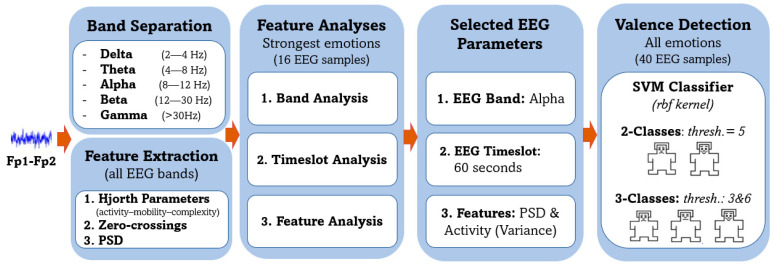
Experimental workflow.

In the present study, an EEG-based subject-dependent valence emotion recognition approach is presented using the difference Fp1-Fp2 signal. Figure 8 illustrates the experimental workflow adopted in order to develop an efficient and reliable system that is suitable for wearable EEG. Initially, the Hjorth parameters (activity–mobility–complexity), zero-crossing, and PSD features were computed from the different frequency bands. Next, the strongest emotions per subject were considered for the feature analyses in which the EEG bands, timeslots, and features were determined. Finally, the selected feature set was used for the binary and multiclass valence emotion classification of the complete DEAP dataset. Since a subject dependent approach was adopted in this work, all the classification experiments were repeated for each of the 32 subjects in the DEAP dataset, and the average accuracies of all subjects were reported as the final performance measure.

KNN and SVM classifiers are the most commonly used for EEG emotion recognition [66,72]. The kNN classifier has the advantages of being simple while giving reliable results [45]. The SVM classifier can be easily tuned for optimal performance. A kNN classifier was used for the feature analyses, whereas both the kNN and SVM with radial basis function (rbf) were considered in the final classification experiments. For the kNN classifier, several k values were compared, then k = 5 was chosen as it was found to give better overall performance. For all cases, the Euclidian distance was considered within the kNN classifier to determine the nearest neighbors. As for the SVM classifier, the hyperparameters (cost and gamma) were repeatedly tuned for each subject in the different experiments using Bayesian optimization. A leave-one-out cross-validation (LOOCV) was used in all the experiments. All feature computations and classification experiments were performed using MATLAB R2021a on an Intel Core i7-5500U CPU @2.4 GHz with 16 GB of RAM. 

### 4.1. Feature Analyses

In this work, the aim of the feature analyses was to determine the most relevant (1) frequency band (delta–theta–alpha–beta–gamma), (2) timeslot (first 20 s–middle 20 s–last 20 s–complete 60 s), and (3) features (activity–mobility–complexity–zero-crossings–PSD) for EEG valence recognition. Sixteen videos per subject were included in the feature analyses, those being the ones with eight highest and eight lowest self-rated valence emotions. Considering only the strongest emotions assures significant discrepancy between the two emotional classes (high valence and low valence) for more reliable feature analyses. A similar approach was previously considered in [52,53].

**A.**  **Band/Feature Analysis**

**Figure 9:**
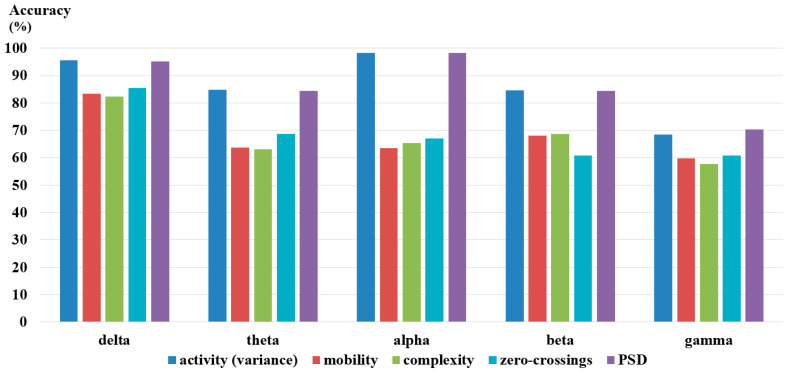
Valence classification accuracies for the different features and EEG frequency bands.

Feature/band analysis was performed in order to determine the frequency bands and features most suitable for valence classification. The three Hjorth parameters, zero-crossings, and PSD features were calculated from the five EEG frequency bands (delta, theta, alpha, beta, gamma). KNN classifier was then used to classify the 1 minute trials into high or low valence. Figure 9 summarizes the valence (happy/sad) classification performance for the different experiments. For all the EEG frequency bands, the variance (Hjorth activity) and PSD were found to result in the highest accuracies. Roshdy et al. [101] have previously shown that the standard deviation, which is the square root of the variance, was highly correlated with valence emotion. PSD is among the most widely accepted measure for valence recognition in the literature [102]. Results of the feature analysis are thus in agreement with previous literature.

Table 3 summarizes the variance and PSD accuracies for the five different frequency bands. Results indicate that for both features, the alpha band gave the most reliable performance closely followed by the delta band. These results are in agreement with several research that showed that the alpha [32,72] and delta [85] bands were relevant for valence emotion detection. The low accuracies attained by the gamma band features were however unconventional as the gamma band was previously shown to be suitable for emotion recognition [55,87]. The gamma band was thus further divided into three subbands which are 30–40 Hz, 40–50 Hz, and 50–60 Hz, and the previous analysis were repeated. Results summarized in Table 4 indicate a significant improvement in performance when the gamma band was subdivided into three different subbands. Best results were attained by the fast gamma subband (50–60 Hz) for which accuracies of 99.02% and 98.63% were achieved for the variance and PSD, respectively, by that outperforming results attained by the same features for the delta and alpha bands. 

Based on the feature/band analysis, it can be deduced that the variance (Hjorth activity) and PSD calculated from the delta, alpha, and fast gamma frequency bands result in the most consistent performance. Further experiments performed in this study will thus only use the indicated features and frequency bands.

**B.**  **Time Slot Analysis**

In the DEAP dataset, a 1 minute EEG recording is provided for each video stimulus per subject. Several previous works considered only the middle time slot omitting the first part for emotions to settle and the last part for fatigue [56,58]. Others used only the last thirty seconds under the assumption that it yields better results [53,67]. In order to test these presumptions, the variance (Hjorth activity) and PSD features were calculated from the first, middle, and last 20 seconds (s) of the EEG recordings for the delta, alpha, and fast gamma bands. Valence classification results for the three indicated timeslots in comparison to using the complete 1 minute are summarized in Table 5. Overall, better valence classification performance is achieved by the alpha and fast gamma bands (~97–99%) than for the delta band (~95–96%). For the delta band, results from the different slots were somewhat close. However, the first timeslot resulted in slightly improved results compared to when the complete 1 minute was considered. As for the alpha and fast gamma bands, results indicate that the middle time slot gave more reliable performance in comparison to the first and last timeslots. Nevertheless, considering the full 1 minute EEG signal resulted in an overall better performance than for any of the 20 s time slots. The full one-minute signal will thus be considered for more consistent performance in all the upcoming experiments.

**C.**  **Feature Boxplots**

At the beginning of this section, the activity (variance), mobility, complexity, zero-crossings, and PSD features were computed from the five EEG frequency bands. Classification results considering the strongest emotions showed that the variance and PSD were the most relevant for valence emotion recognition regardless of the frequency band. Specifically, experimentation results showed that the variance and PSD computed from the delta, alpha, and fast gamma full 1 minute EEG signals resulted in the most reliable valence emotion classification performance in comparison to the other considered cases.

**Figure 10:**
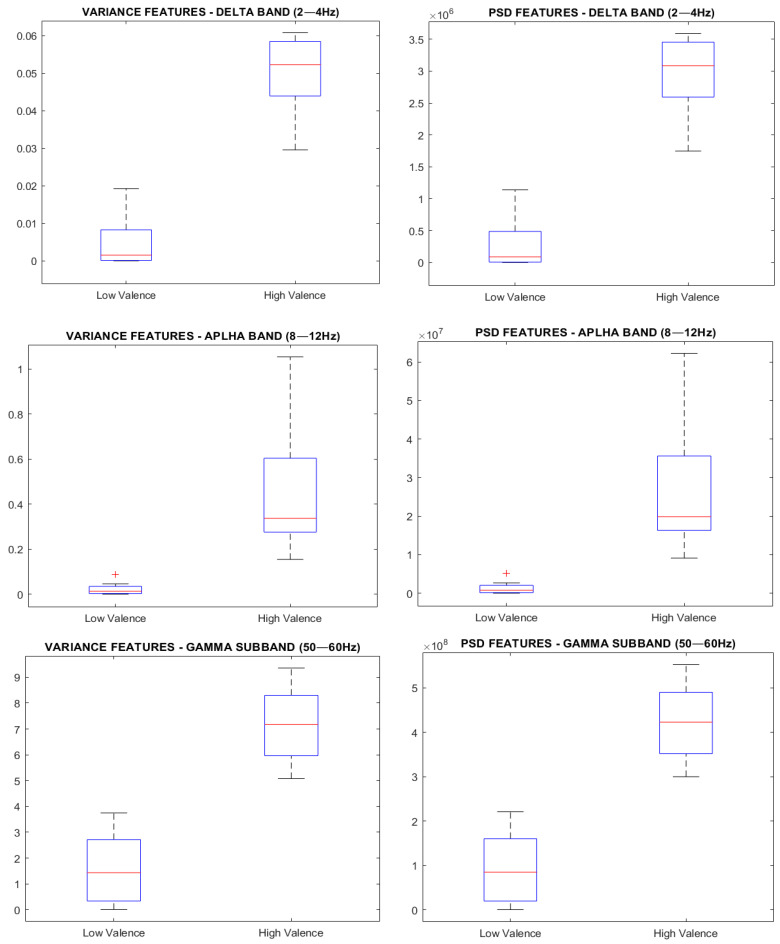
Boxplots of the variance and PSD features for the delta, alpha, and fast gamma bands considering the full 1 minute EEG signal.

In this subsection, the boxplots of the variance and PSD were generated (Figure 10) to illustrate the features’ distributions for the two valence classes: low valence (sad) and high valence (happy). Boxplots display a five-number summary of the data including the minimum, first quartile, second quartile (median), third quartile, and maximum. For both features, the boxplots demonstrate significant discrepancy between the two valence classes which emphasizes their relevance as previously shown in the different classification experiments within the previous subsections.

### 4.2. Valence Classifications

In this section, the subject dependent valence emotion classifications were performed considering all the forty video trials included in the DEAP dataset. The variance (Hjorth activity) and PSD features were computed from the full 1 minute delta, alpha, and fast gamma bands which were found in the previous section to be the most relevant for valence classification. Variance and PSD were used both individually and collectively and results were given for each case. KNN and SVM with rbf kernel were considered in all experiments. 

Table 6 and Table 7 summarize the binary classification accuracies for the kNN and SVM classifiers, respectively. Overall, the SVM classifier gave better accuracies than the kNN classifier. The alpha band is shown to give consistently better results closely followed by the delta band, whereas the fast gamma band results are almost 10% less for both classifiers. Fast gamma is thus shown to be reliable when discriminating between strong sad and happy emotions attaining accuracies that were as high as 99% (Table 4), yet less useful when more mellow emotional states were additionally involved. 

Generally, variance (Hjorth activity) and PSD gave close results in all experiments. For the alpha and delta bands, all achieved accuracies were greater than or equal to 95%, indicating the efficacy of the considered features for valence emotional recognition. Variance did, however, give slightly better results than PSD in most cases. Combining these two features resulted in an overall more consistent performance. Best results were achieved when the combined features were calculated from the alpha band resulting in accuracies of 96.33% and 97.42% for the kNN and SVM classifiers, respectively. Several research has shown that the frontal channels’ alpha band was significantly affected by a person’s happiness and sadness emotions [28,103]. The findings of this work, in which the alpha band was found to be more reliable than other frequency bands for valence recognition, are thus in agreement with previous literature.

For the sake of attaining a more comprehensive insight on the performance of the proposed method, the valence classification accuracies per subject for the combined variance and PSD features for the delta, alpha, and fast gamma bands are presented in Table 8. For the alpha band, twenty-eight and thirty of the total thirty-two DEAP subjects had their emotions recognized with an accuracy that is greater than or equal to 95% for the kNN and SVM classifiers, respectively, which indicates the reliability of the considered features. 

In order to further investigate these results, the median, average, and standard deviation of the valence ratings of the two subjects with the lowest and highest SVM accuracies in the alpha band were inspected and summarized in Table 9. Furthermore, these statistical measures were also calculated for all the thirty-two subjects in the DEAP dataset. For subject #27 (one of the subjects with the lowest accuracies), it is noticed that both the median and average of the valence ratings are higher than the value of the threshold considered in this work for the low/high valence class separation. Modifying this threshold value to become six instead of five, which is closer to subject #27′s median and average, indeed resulted in improving this subject’s emotional recognition accuracy by 5% to become 97.5%. On the other hand, the increased threshold had no effect or minimal effect on the other considered subjects and minimal effect on the overall performance. These results indicate the robustness of the two implemented measures for valence emotion recognition whilst also highlighting the importance of considering subject variability for more reliable results. 

Table 10 summarizes the three-class valence classification results using the variance, PSD, as well as both features calculated from the delta, alpha, and fast gamma bands. Similar to the binary classifications, best results were attained when the features were computed from the alpha band, closely followed by the delta band. For the alpha and delta bands, considering one of the features or both combined resulted in close accuracies ranging from 94.22% to 95.39%. Best performance (accuracy = 95.39%) was attained when the variance was computed from the alpha band.

## 5. Discussion

In the present study, an efficient EEG-based valence recognition method was presented that considers only the difference Fp1-Fp2 signal for feature extraction. Analyses showed that the variance and PSD computed from the 1 minute alpha band were the most suitable for valence recognition. Final classification experiments considering the entire DEAP dataset resulted in accuracies of 97.42% and 95.39% for the two and three class valence classifications, respectively. Torres et al. [72] have reported that in previous literature, accuracies were on average about 85% and 68% for two and three class EEG-based valence classifications, respectively. The performance of the proposed methods thus surpasses the average performance of EEG-based valence detection methods by approximately 10% and 27% for two- and three-class classifications, respectively, indicating the superiority of the implemented method.

The notion that few simple handcrafted features can give promising results in EEG-based valence classification has been previously demonstrated in several research papers. In an early work by Sourina et al. [104], accuracies well above 90% were achieved for all subjects considering only three frontal channels using music to invoke the emotional stimuli. In another work by Amin et al. [105], emotion recognition accuracies exceeding 98% were attained considering only the relative wavelet energy, which was calculated from the delta band of 128 electrodes. However, for both these works performance could not be compared to other methods as private datasets were utilized. A later work by Thejaswini et al. [32] achieved an overall average accuracy of 91.2% upon classifying the SEED dataset to three classes: positive, neutral, and negative emotions. They implemented simple statistical features including the RASM and Hjorth parameters, but again considering twenty-seven electrode pairs for the feature computations.

The DEAP dataset, considered in this study, is reportedly the most widely utilized for EEG emotional recognition [72] which facilitates comparison between the different approaches. Table 11 summarizes the performance of several other EEG emotion recognition methods from literature that also used the DEAP dataset. The comparison indicates the EEG channels and frequency bands considered in each approach, as well as the binary classification accuracy. For the sake of a fair comparison, all valence emotion recognition methods included are based on subject-dependent experiments, which is the approach considered in this work. Wu et al. [53], like in this work, used only the FP1 and Fp2 frontal channels, yet achieved a relatively low accuracy of 75.18%. Other methods used all the EEG channels whether individually or in the form of channel pairs. In addition, most of the studies summarized in Table 11 considered all the frequency channels by that ignoring the significance of some bands over others for valence emotion recognition. Overall, the valence classification accuracies of the summarized approaches mostly range from 75.18% to 96.65%. The EEG valence emotion recognition method introduced in the present study results in an accuracy of 97.42% by that outperforming several state-of-the-art methods deep learning methods. 

Nevertheless, the recent approach introduced by Cheng et al. [82], which is based on randomized CNN and ensemble learning, resulted in an overall accuracy of 99.17% which is 1.75% higher than the implemented method. In their work, they reported an average training time of 35.15 s. As for the proposed method, an average of 0.06 s were required for the feature computation, training, and classification. Nevertheless, the machine learning-based proposed approach, even though performing not as well as Cheng et al.’s method, has the valuable merit of being simpler to reproduce. 

The proposed EEG-based valence emotion recognition method was shown to result in reliable performance while relying on statistical measures that are simple to compute. In addition, it relies on standard machine learning algorithms that are easily configured. No image construction was required, and no complex neural networks needed to be trained. In the literature, several works have also shown that handcrafted features can achieve comparable performance to deep learning approaches with the former having the merit of reduced computational complexity which could be attractive in real-time applications [106,107,108]. Another advantage of the presented method is that unlike in other literature where all the frequency bands or the raw EEG signal were considered, only the alpha band was used for feature extraction. The alpha band was utilized in this work as it was shown in the analyses performed in Section 4.1 to be the most relevant for valence detection. Interestingly, several clinical studies have previously shown that there is indeed a relationship between the alpha activity measured from the prefrontal cortex and emotional response [109,110].

The proposed method considers only the Fp1-Fp2 channel pair from which the alpha band’s variance and PSD were computed, by that minimizing the computational overhead whilst achieving reliable performance making it suitable for wearable EEG headsets used in real-time applications [26,111]. Overall, the results attained here are quite promising. Yet, there is still room for enhancement of the suggested method. Future work includes considering arousal along with valence recognition, as well as calculating other statistical features that are relevant to EEG-based emotion recognition such as entropy and RASM. In addition, the integration of handcrafted and deep features can be investigated. Explainable AI (XAI) methods can then be implemented to understand what the models are learning and why the specific decisions were made. XAI can also be applied to investigate whether EEG-based emotion detection is gender or culture dependent, as is speech emotion recognition [112].

## 6. Conclusions

EEG-based subject-dependent valence emotion recognition is widely implemented in personalized emotion AI applications. In this work, the difference signal (Fp1-Fp2) was used to calculate the Hjorth parameters (variance-mobility-complexity), zero-crossings, and PSD features for the emotional valence detection using the benchmark DEAP dataset. Several analyses were performed to determine the features, frequency band, and timeslot most suitable for reliable subject-based valence recognition. Primarily, only the eight strongest high and low valence emotions per subject were considered for analysis to assure significant discrepancy between the two classes. Classification results indicated that the variance and PSD features were the most suitable for valence recognition regardless of the considered frequency channel. Nevertheless, the delta, alpha, and fast gamma bands were shown to be the most relevant for valence recognition. Boxplots of the variance and PSD features for the most relevant frequency bands validated and supported the classification results. In addition, calculating the features from the complete 1 minute EEG signal was found to give more reliable performance than when only a 20 s timeslot was used for feature computation. Best results were achieved when the variance and PSD were computed from the alpha band resulting in accuracies of 97.42% and 95.0% for the binary and multiclass classification, respectively. Comparison to previous literature showed that implemented method outperformed several state-of-the-art approaches with the advantage of reduced computational complexity due to the reduced number of electrodes, features, and frequency bands considered. This approach would thus be highly attractive for practical EEG-based emotion AI systems relying on wearable EEG devices.

## Figures and Tables

**Table 1:** Characteristics of the five basic brain waves.

Band	Symbol	Frequency Range	Psychological State	
**Delta**	*Δ*	<4 Hz	unconsciousness	Deep sleep
**Theta**	*θ*	4–8 Hz	subconsciousness	Light sleep and meditation
**Alpha**	*α*	8–12 Hz	consciousness	Normal relaxed yet alert adult
**Beta**	*β*	12–30 Hz		Daily activities
**Gamma**	*δ*	>30 Hz		Complex brain activities

**Table 2:** Summary of EEG-based emotion recognition approaches that utilize the DEAP dataset.

Research Paper	Channels	EEG Bands	Features	Classifier	Dep./ Indep.	Val./ Arl.	Acc. %
Mohammadi et al., 2017 [55]	Fp1, Fp2	Gamma	Wavelet Features	kNN	Indep.	Val. Arl.	80.68 74.60
	Fp1, Fp2, F7, F8, F3, F4, FC5, FC6, FC1, FC2					Val. Arl.	86.75 84.05
Salma et al., 2017 [59]	All	Raw	Deep Features (LSTM)	Sigmoid	Dep.	Val. Arl.	85.45 85.65
Wu et al., 2017 [53]	Fp1, Fp2	All	Frequency, WT Features	GBDT	Dep.	Val.	75.18
Zhuang et al., 2017 [76]	FP1, FP2, F7, F8, T7, T8, P7, P8	Beta, Gamma	Time (EMD)	SVM	Dep.	Val. Arl.	69.10 71.99
Eun et al., 2018 [77]	Fp1, Fp2, F3, F4, T7, T8, P3, P4^*^	Raw	Deep Features (LSTM)	Sigmoid	Indep.	Val. Arl.	78.00 74.65
Putra, 2018 [75]	All	All except delta	Wavelet Features	kNN	Dep.	Val. Arl.	59.00 65.70
	All	All except delta	Wavelet Features	kNN	Indep.	Val. Arl.	58.90 64.30
Yang et al., 2018 [60]	All	Raw	Deep Features (LSTM, CNN)	Softmax	Dep.	Val. Arl.	90.80 91.03
Parui et al., 2019 [36]	All	Raw	Time, WT Features	XGBoost	Indep.	Val. Arl.	75.97 74.20
		All	Frequency Features				
Xing et al., 2019 [78]	All	All except delta	Frequency Features	LSTM	Indep.	Val. Arl.	81.10 74.38
Cui et al., 2020 [79]	Symmetric Channels	All except delta	Regional-Asymmetric CNN (RACNN)	Softmax	Dep.	Val. Arl.	96.65 97.11
Garg and Verma, 2020 [65]	All	Raw	Scalogram Images	GoogleNet (pretrained)	Indep.	Val. Arl.	92.19 61.23
Nath et al., 2020 [73,80]	All	All	Band Power	LSTM	Dep.	Val. Arl.	94.69 93.13
				SVM	Indep.	Val. Arl.	72.19 71.25
Aslan, 2021, [62]	All	Raw	Scalogram Images	GoogleNet (pretrained) +SVM	Indep.	Val. Arl.	91.20 93.70
Ozdemir et al., 2021 [81]	All	Alpha, Beta, Gamma	Multi-Spectral Topology Images	CNN, LSTM + Softmax	Indep.	Val. Arl.	90.62 86.13
Huang, 2021 [61]	Symmetric Channels	Raw signal	Bi-hemisphere spatial features	CNN	Dep.	Val. Arl.	94.38 94.72
					Indep.	Val. Arl.	68.14 63.94
Yin et al., 2021 [48]	All	Raw signal	Differential Entropy Cube	GCNN, LSTM	Dep.	Val. Arl.	90.45 90.60
	All				Indep.	Val. Arl.	84.81 85.27
Zhang et al., 2021 [58]	Fp1, Fp2, F3, F4, AF3, AF4^*^	All	Time, Frequency	Softmax	Indep.	Val. Arl.	84.71 83.28
		Raw signal	Deep Features (HFCNN)				
Cheng et al., 2022 [82]	All	Raw Signal	Deep Features (randomized CNN)	Ensemble	Dep.	Val. Arl.	99.19 99.25
Gao et al., 2022 [37]	All	All except delta	Time, Frequency Features	CNN + SVM	Indep.	Val. Arl.	80.52 75.22

**Table 3:** Valence classification accuracies (%) for the different EEG bands using activity and PSD.

	All (2–60 Hz)	Delta (2–4 Hz)	Theta (4–8 Hz)	Alpha (8–12 Hz)	Beta (12–30 Hz)	Gamma (30–60 Hz)
**variance**	62.50	**95.51**	84.77	**98.24**	84.57	68.56
**PSD**	61.33	**95.12**	84.38	**97.85**	84.38	70.31

**Table 4:** Valence classification accuracies (%) for the different gamma subbands using activity and PSD.

	30–60 Hz	30–40 Hz	40–50 Hz	50–60 Hz
**variance**	68.56	91.99	91.40	**99.02**
**PSD**	70.31	91.99	91.02	**9.63**

**Table 5:** Strongest emotion classification accuracies (%) for different EEG time slots.

	Delta (2–4 Hz)		Alpha (8–12 Hz)		Gamma (50–60 Hz)	
	Variance	PSD	Variance	PSD	Variance	PSD
**1–20 s**	96.29	96.09	97.46	97.07	97.66	97.66
**20–40 s**	95.51	95.70	97.46	97.46	98.05	98.05
**40–60 s**	94.92	95.51	96.68	97.27	98.05	97.85
**1–60 s**	**95.51**	**95.12**	**98.24**	**98.24**	**99.02**	**98.63**

**Table 6:** Valence classification accuracies (%) for the complete DEAP dataset (kNN).

	Delta (2–4 Hz)	Alpha (8–12 Hz)	Fast Gamma (50–60 Hz)
**Variance**	95.08	96.09	85.23
**PSD**	95.08	96.25	84.76
**Variance + PSD**	95.00	**96.33**	85.55

**Table 7:** Valence classification accuracies (%) for the complete DEAP dataset (SVM-rbf).

	Delta (2–4 Hz)	Alpha (8–12 Hz)	Fast Gamma (50–60 Hz)
**Variance**	96.95	97.26	87.58
**PSD**	95.55	96.80	87.50
**Variance + PSD**	97.19	**97.42**	87.11

**Table 8:** Valence classification accuracies (%) per subject for the combined variance and PSD features considering the complete DEAP dataset.

Subject	kNN			SVM (rbf)		
	Delta	Alpha	Fast Gamma	Delta	Alpha	Fast Gamma
1	95.0	97.5	77.5	97.5	**97.5**	77.5
2	92.5	95.0	77.5	87.5	**95.0**	82.5
3	95.0	97.5	72.5	97.5	**97.5**	75.0
4	100	92.5	60.0	97.5	**95.0**	72.5
5	95.0	90.0	90.0	97.5	**95.0**	92.5
6	100	95.0	97.5	100	**97.5**	95.0
7	95.0	95.0	87.5	97.5	**100**	92.5
8	95.0	97.5	77.5	97.5	**100**	85.0
9	95.0	97.5	82.5	97.5	**97.5**	87.5
10	95.0	97.5	90.0	100	**95.0**	90.0
11	90.0	92.5	90.0	90.0	**95.0**	87.5
12	95.0	100	85.0	100	**100**	85.0
13	97.5	95.0	70.0	100	**97.5**	67.5
14	95.0	97.5	97.5	100	**97.5**	100
15	95.0	97.5	82.5	97.5	**97.5**	82.5
16	100	95.0	75.0	100	**95.0**	77.5
17	95.0	97.5	85.0	97.5	**100**	85.0
18	97.5	100	90.0	97.5	**100**	92.5
19	95.0	95.0	95.0	97.5	**92.5**	95.0
20	95.0	97.5	85.0	97.5	**97.5**	90.0
21	95.0	97.5	90.0	100	**97.5**	97.5
22	97.5	100	90.0	100	**100**	85.0
23	92.5	100	95.0	95.0	**100**	95.0
24	97.5	97.5	77.5	95.0	**97.5**	77.5
25	95.0	95.0	87.5	97.5	**97.5**	85.0
26	95.0	95.0	100	97.5	**97.5**	100
27	97.5	90.0	95.0	100	**92.5**	95.0
28	87.5	97.5	97.5	90.0	**97.5**	97.5
29	95.0	95.0	97.5	97.5	**95.0**	97.5
30	95.0	97.5	87.5	97.5	**100**	90.0
31	85.0	97.5	82.5	92.5	**100**	85.0
32	95.0	97.5	70.0	100	**100**	70.0
**Average**	**95.0**	**96.33**	**85.55**	**97.19**	**97.42**	**87.11**

**Table 9:** Valence ratings statistical measures and classification accuracies for different valence thresholds, given for the subjects with the lowest and highest performance as well as for the complete DEAP dataset.

		Highest Accuracies		Lowest Accuracies		All Subjects
		Subject #12	Subject #22	Subject #19	Subject #27	
**Valence ratings statistical measures**	**Median**	5.04	5.00	5.04	6.08	5.04
	**Average**	4.88	4.69	5.23	6.08	5.25
	**Std. deviation**	2.24	2.44	1.80	2.18	2.13
**Accuracies** **(SVM)**	**Threshold = 5**	100	100	92.5	92.5	97.42
	**Threshold = 6**	97.5	100	92.5	97.5	96.56

**Table 10:** Valence three-class accuracies (%) for the complete DEAP dataset (SVM-rbf).

	Delta (2–4 Hz)	Alpha (8–12 Hz)	Fast Gamma (50–60 Hz)
**Variance**	94.30	**95.39**	78.13
**PSD**	94.69	94.22	78.28
**Variance + PSD**	94.92	**95.00**	78.44

**Table 11:** Valence (happy/sad) classification performance for the DEAP dataset.

Method	Year	Method	Channels	Bands	Acc. %
Wu et al. [53]	2017	FFT and WT features with GBDT	Fp1, Fp2	All	75.18
Salma et al. [59]	2017	LSTM and RNN	All	Raw	85.45
Yang et al. [60]	2018	LSTM and CNN	All	All	90.80
Cui et al. [79]	2020	Differential Entropy + SVM	Symmetric channel pairs	All except delta	89.09
		Multilayer Perceptron (MLP)			92.57
		Regional-Asymmetric CNN (RACNN)			96.65
Nath et al. [80]	2020	Band power with LSTM	All	All	94.69
Yin et al. [48]	2021	Differential entropy with ECLGCNN	All	All	80.52
Huang et al. [61]	2021	Bi-hemisphere discrepancy CNN	Symmetric channel pairs	Raw	94.38
Chen et al. [82]	2022	Ensemble Deep Randomized-CNN	All	Raw	99.19
Proposed	2022	Variance + PSD with SVM	Fp1-Fp2	Alpha	97.42

## Data Availability

The DEAP dataset supporting reported results is a public dataset that can be found here: https://www.eecs.qmul.ac.uk/mmv/datasets/deap/download.html, accessed on 16 January 2023.
